# Posterior short segment fixation including the fractured vertebra combined with kyphoplasty for unstable thoracolumbar osteoporotic burst fracture

**DOI:** 10.1186/s12891-020-03576-9

**Published:** 2020-08-21

**Authors:** Xudong Hu, Weihu Ma, Jianming Chen, Yang Wang, Weiyu Jiang

**Affiliations:** Department of Spine Surgery, Ningbo No.6 Hospital, Zhongshan East Road 1059#, Ningbo, 315040 Zhejiang Province China

**Keywords:** Thoracolumbar burst fracture, Osteoporotic, Posterior shout segment fixation, Kyphoplasty

## Abstract

**Background:**

Various studies have described the efficacy and safety of the treatment for unstable thoracolumbar osteoporotic burst fracture, however, there is still no consensus on the optimal treatment regimen. The aim of this study was to evaluate the clinical and radiographic results of posterior short segment fixation including the fractured vertebra (PSFFV) combined with kyphoplasty (KP) for unstable thoracolumbar osteoporotic burst fracture.

**Methods:**

Forty-three patients with unstable thoracolumbar osteoporotic burst fracture underwent PSFFV combined with KP from January 2015 to December 2017 were analyzed retrospectively. Visual Analogue Scale (VAS) and Oswestry Disability Index (ODI) was used to evaluate the clinical outcome, radiological parametres including local kyphotic Cobb angle, percentage of the anterior, middle and posterior height of the fractured vertebra were measured and compared pre-operation, post-operation and at final follow-up.

**Results:**

All patients underwent surgery successfully and with an average follow-up of 19.2 ± 6.7 months (rang 15–32). The VAS decreased from 7.1 ± 2.3 pre-operation to 1.6 ± 0.4 at the final follow-up (*p* < 0.05). The ODI decreased from 83.1 ± 10.5 pre-operation to 19.2 ± 7.3 (*P* < 0.05) at the final follow-up. The correction of local kyphotic angle was 16.9° ± 5.3° (*p* < 0.05), and the loss of correction was 3.3° ± 2.6° (*p* > 0.05), the correction of anterior vertebral height was 30.8% ± 8.6% (*p* < 0.05), and the loss of correction was 4.5% ± 3.9% (*p* > 0.05), the correction of middle vertebral height was 26.4% ± 5.8% (*p* < 0.05), and the loss of correction was 2.0% ± 1.6% (*p* > 0.05), the correction of posterior vertebral height was 9.4% ± 6.9% (*p* < 0.05), and the loss of correction was 1.6% ± 1.3% (*p* > 0.05). Two cases of screw pullout and 8 cases of cement leakage were observed, but without clinical consequence.

**Conclusions:**

PSFFV combined with KP is a reliable and safe procedure with satisfactory clinical and radiological results for the treatment of unstable thoracolumbar osteoporotic burst fracture.

## Background

The incidence of osteoporotic vertebral fractures (OVFs) increased rapidly over the past years because of the aging population, ballon kyphoplasty (KP) has been proved to be safe and effective for most of the osteoporotic vertebral compression fractures [1, 2]. However, there is still no consensus on the optimal treatment regimen for osteoporotic vertebral burst fracture, KP has been reported to be an effective, low-risk procedure for osteoporotic vertebral fracture with partial inclusion of the posterior wall of the vertebral body, but risks is still inevitable, such as cement leakage to the spinal canal, unsatisfactory fracture reduction, progressive kyphosis and secondly neurological impairment, some patiensts even need revision surgery [3].

Posterior pedicle screw fixation has been one of the most highly regarded techniques for treating unstable thoracolumbar burst fractures over the past decade, and some studies introduced 4-screws fixation immediately adjacent to the fractured vertebral combined with cement augmentation for the treatment of osteoporotic vertebral burst fracture, the overall result was acceptable, however, loss of reduction was unavoidable [4]. Clinical and biomechanical studies confirmed addition pedicle screw fixation in fractured vertebral significantly improve the stability compared to conventional 4-screws fixation for thoracolumbar burst fracture with normal bone mineral density [5, 6]. So we hypothesis that posterior short segment fixation including the fractured vertebra (PSFFV) combined with KP provide stronger fixation for unstable thoracolumbar osteoporotic burst fracture, and the aim of this study was to evaluate the feasibility and efficacy of this procedure.

## Methods

### Demographic data

This study was approved by institutional review board of the ethics committee of our institute and all patients provided informed consent for the procedure. 43 patients from January 2015 to December 2017 were enrolled in this retrospective study on the basis of the following criteria (Table 1). Inclusion criteria: (1) a single-leve fracture, (2) a fracture at the levels of T11-L3, (3) fracture caused by low-energy trauma with osteoporosis (T score ranged from − 2.5 to − 3.5), (4) a type A3 or A4 burst fracture according to the AO classification [7], (5) fracture without neurological symptoms, (6) the bilateral vertebral pedicles were intact of the fractured vertebra. Exclusion criteria: (1) old fracture, (2) metastatic fracture. Patients comprised of 31 females and 12 males with mean age of 62.4 ± 7.5 years (range: 59–76). All of the patients were injuried by slipping down. The fracture level was T11 in 5 patients, T12 in 10 patients, L1 in 16 patients, L2 in 8 patients, L3 in 4 patients. Twenty-three patients were type A3 and 20 patients were type A4 fractures according AO classification.

**Table 1:** Demographic data

Characteristic	No. Patients
**Sex (n, %)**	
Male	12 (27.9%)
Female	31 (72.1%)
**Ages (year)**	62.4 ± 7.5
**Level of fractures (n, %)**	
T11	5 (11.6%)
T12	10 (23.3%)
L1	16 (37.2%)
L2	8 (18.6%)
L3	4 (9.3%)
**Type of fractures (n, %)**	
A3	23 (53.5%)
A4	20 (46.5%)

### Surgery procedure

After general tracheal anesthesia, the patient was placed in a prone position, the fractured and adjacent veterbal pedicles were determined under the C-arm fluoroscopy. A midline incision was performed, and the pedicle screw insertion point was exposed through the paraspinal sacrospinalis muscle-splitting approach, the bilateral pedicle screws adjacent to the fractured vertebra and unilateral pedicle screw of the fractured vertebra were inserted through freehand technique (Kang peng, China), the angle, diameter and length of the screws were determined according to the pre-operative CT scan. Two rods with appropriate length were sited and attached to the screws, the fracture was reduced by screw-rod distraction. Cannula was positioned into the central of the fractured vertebra body through the unscrewed pedicle by the transpedicular technique under fluoroscopic guidance, the balloon was then placed into the vertebral body through the cannula, and the vertebral was reduced via ballon infalting, then cement was injected into the vertebral body, this was performed slowly under fluoroscopic control until 6.0 ml of cement was instilled or signs of cement leakage was visible. The rod on the side of no screw in the fractured vertebral was removed, and pedicle screw was inserted, then the rod was reconnected and tightened. Two negative pressure drainages were implemented and the incision was closed.

### Postoperative management

Antibiotics were routinely applied 24 h post-operation and anti-osteoporosis treatment was administrated routinely, the drainages were removed when it was less than 50 ml/day, and the patients started to get out of bed with the protection of a brace, which should be lasted 3 months post-operation.

### Clinical assessment

For all patients, the following clinical index were observed, including the operation time, amount of surgical blood loss, amount of cement instillation, number of cement leakage, hospitalization time, complications and surgical revisions. Visual analog scale (VAS) was used to evaluate back pain and Oswestry disability index (ODI) was used to evaluate the functional outcome at differnet times before surgery and postoperatively.

### Radiological assessment

Plain radiographs were obtained pre-operation, immediately post-operation, and at the final follow-up. The following parameters were observed and analysed: (1) the local kyphotic angle, it was defined as the angle measured between the superior endplate of the upper vertebra and the inferior endplate of the lower vertebra. (2) percentage of the anterior, middle and posterior height of the fractured vertebra. The normal anterior, middle and posterior height of the fractured vertebra was determined by averaging the anterior, middle and posterior heights of the adjacent upper and lower vertebra. The relative anterior, middle and posterior height of the fractured vertebra was defined as the anterior, middle and posterior height of the fractured vertebra divided by the normal anterior, middle and posterior height of the vertebra, and all of them were expressed as a percentage.

### Statistical analysis

Datas were presented as the means ± standard deviations and analysed by SPSS software (version 20.0; SPSS Inc., Chicago, IL, USA). A paired Student’s t-test and the Wilcoxon nonparametric test were used to evaluate data changes at different times. A value of less than 0.05 was considered statistical significance.

## Results

All patients underwent the surgery successfully as planed and with an average follow-up of 19.2 ± 6.7 months (rang 15–32). The mean operation time was 83.2 ± 23.5 min (rang: 72–108), the mean blood loss was 120.5 ± 50.6 ml (rang: 95–180), the mean instillation of cement was 4.6 ± 1.7 ml (rang: 2.8–6), cement leakage was observed in 8 (18.7%) cases, 3 cases at the adjacent discal space, 3 cases at the anterior of the fractured vertebra and 2 cases at lateral, none of them had clinical consequence, the mean hospitalization stay days was 6.3 ± 2.2 (rang: 5–9). There was no infection and no neurological complications, two cases of screw pullout were found at the final follow-up but without revision surgery.

The mean back pain score (VAS) decreased from 7.1 ± 2.3 preoperatively to 2.3 ± 1.1 at discharge (*p* < 0.05), and it was 1.6 ± 0.4 at the final follow-up (*p* < 0.05). The ODI decreased from 83.1 ± 10.5 preoperatively to 19.2 ± 7.3 at the final follow-up (*P* < 0.05).

**Fig. 1:**
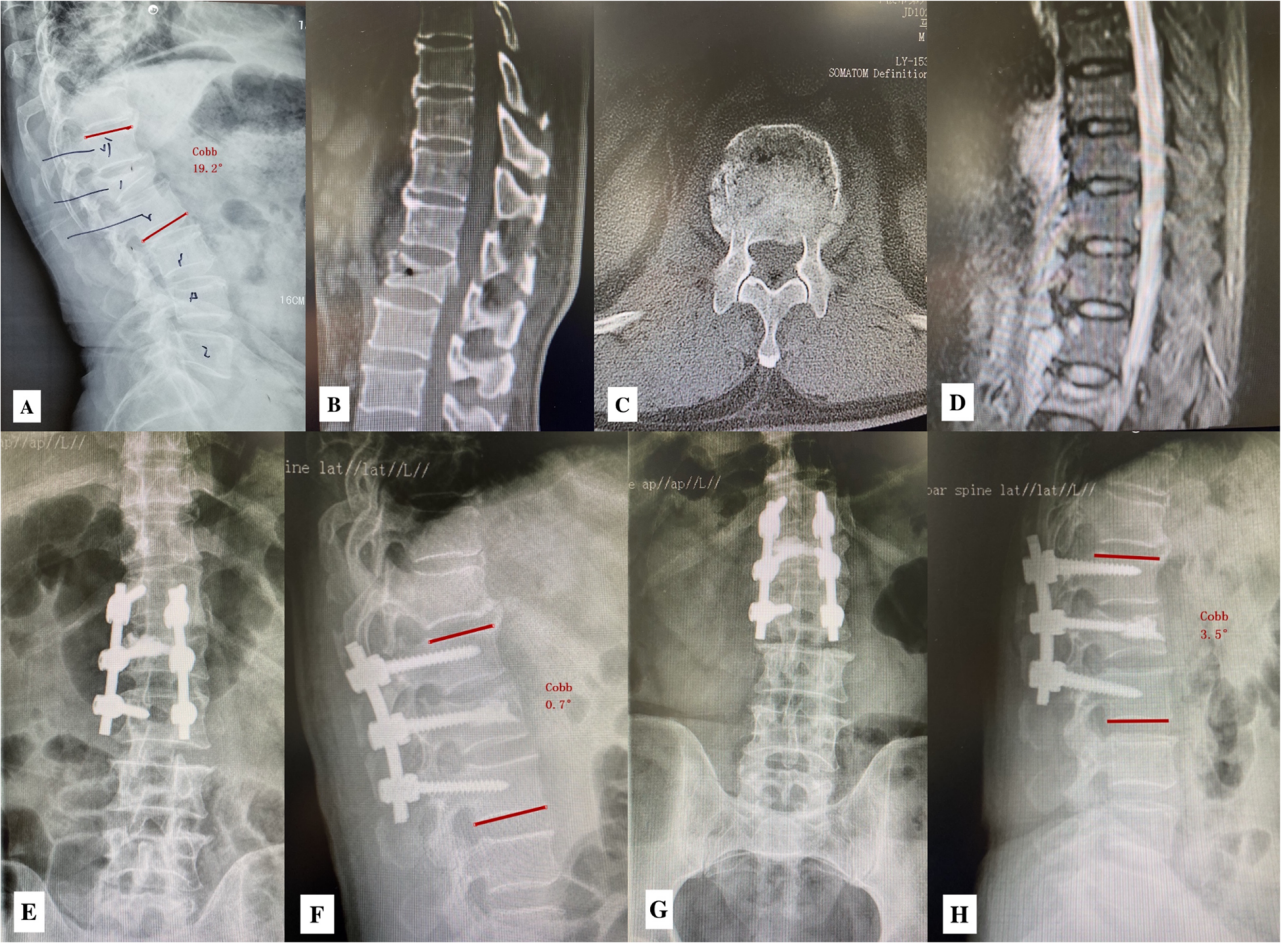
A 70-year-old male patient with L1 osteoporotic burst fracture underwent PSFFV combined with KP. **a**-**d** The preoperative X-ray, CT scan and MRI showed L1 fresh burst fracture, the Cobb angle was 19.2°, the anterior, middle and posterior vertebral height was 38, 42 and 72% respectively. **e**, **f** Postoperative X-ray showed the fractured vertebral was restored, the correction of local kyphotic angle was 18.7°, and the anterior, middle and posterior vertebral height was almost normal. **g**, **h** The final follow-up X-ray showed the correction was greatly maintained, the kyphosis was only 2.8°, and the anterior, middle and posterior vertebral height was 92, 89 and 96% respectively

The Cobb angle and loss rates of vertebral height preoperatively, postoperatively and at the final follow-up period were shown in Table 2. The correction of local kyphotic angle was 16.9° ± 5.3° (*p* < 0.05), and the loss of correction was 3.3° ± 2.6° (*p* > 0.05), there was still 13.6° correction from the time of injury to the final follow-up (*p* < 0.05). The correction of anterior vertebral height was 30.8% ± 8.6% (*p* < 0.05), and the loss of correction was 4.5% ± 3.9% (*p* > 0.05), there was still 26.3% restoration between the time pre-operation and the final follow-up (*p* < 0.05). The correction of middle vertebral height was 26.4% ± 5.8% (*p* < 0.05), and the loss of correction was 2.0% ± 1.6% (*p* > 0.05), there was still 24.5% restoration between the time pre-operation and the final follow-up (*p* < 0.05). The correction of posterior vertebral height was 9.4% ± 6.9% (*p* < 0.05), and the loss of correction was 1.6% ± 1.3% (*p* > 0.05), there was still 7.8% restoration between the time pre-operation and the final follow-up (*p* < 0.05) (Fig. 1).

**Table 2:** Radiographic parameters of the patients (mean ± standard deviation)

Variable	Local kyphosis (°)	Anterior height (%)	Middle height (%)	Poterior height (%)
Preoperative	23.2 ± 6.1	55.8 ± 12.2	62.3 ± 10.5	82.7 ± 8.4
Postoperative	6.3 ± 3.9	86.6 ± 13.1	88.7 ± 8.3	92.1 ± 7.3
Final follow-up	9.6 ± 4.6	82.1 ± 12.6	86.7 ± 7.8	90.5 ± 8.1
Correction by surgery	16.9 ± 5.3	30.8 ± 8.6	26.4 ± 5.8	9.4 ± 6.9
Loss of correction at final follow-up	3.3 ± 2.6	4.5 ± 3.9	2.0 ± 1.6	1.6 ± 1.3

## Discussion

The optimal treatment for OVFs has been controversial, effectiveness of vertebroplasty (VP) and KP has been confirmed by various studies [8, 9], but the recent evidence based review reported, for the osteoporotic vertebral compression fractures, cement augmentation was effective and safe only in patients with acute fractures having persistent and severe pain, but there was no benefits among patients with older fractures or those bearing non-severe symptoms [10]. DGOU classified OVFs into 5 types according to the morphological patterns and the biomechanical stability of the fractures, and treatment algorithm was introduced for different types, only type 1 and type 2 fracures were recommended for cement augmentation if conservative treatment failed, while the other threes types need short or long posterior instrumentation with an option of cement augmentation or anterior reconstruction [11]. Hence, individualized therapeutic options should be considered for OVFs, while surgical intervention is typically recommended for unstable osteoporotic burst fractures, the purpose is to stabilize the injured segment, rehabilitate the sagittal alignment. However, there is no “gold standard” surgical method for treating unstable osteoporotic burst fracture, a number of surgical options have been described, including cement augmentation alone, cement augmented screw fixation, posterior screw fixation combined with cement augmentation [3, 12–14].

The reasonable surgical procedure should realignment the deformity initially, and the restoration can be maintained for a long-term. Zhong et al. compared the results between VP and VP combined with short segment posterior instrumentation for type 4 OVFs according to DGOU classification, the later was more effective in the correction and maintain of segment height and spine alignment [15]. From the finite element analysis, four segment fixation was suggested for patients with thoracolumbar osteoporotic burst fractures [16]. However, long-segment fixation was associated with multi-disadvantages, including long surgical time, high amounts of blood loss, and the sacrifice of motion. In the present study, we designed PSFFV combined with KP for unstable thoracolumbar osteoporotic burst fracture, and the efficacy was encouraging, the VAS (7.1 vs 1.6) and ODI (83.1 vs 19.2) improved tremendously at the final follow-up compared to preoperatively, the kyphotic angle decreased from 23.2 ± 6.1 pre-operation to 6.3 ± 3.9 post-operation, and the anterior, middle and posterior height of the vertebral was reconstructed from 55.8 ± 12.2 to 86.6 ± 13.1, 62.3 ± 10.5 to 88.7 ± 8.3, 82.7 ± 8.4 to 92.1 ± 7.3 respectively, it was more important that the correction of the Cobb angle and the verbetral height was stable over time, loss of reduction was minimal at the final follow-up.

Posterior short segment fixation including the fractured vertebral has been widely applied for thoracolumbar fractures, this method can reduce the operation time and bloods loss, preserve motion segments compared to long-segment posterior fixation, and provide greater mechanical strength to prevent early implant failure compared with traditional 4-screws short-segment posterior fixation [17]. However, diminished bone quality in osteoporotic vertebral impairs primary screw purchase, and screw loosening incidences will be considerably higher than with normal bone mineral density, cement augmented screw fixation may reduce loosening risks [18]. Kim et al. demonstrated cement augmented six screw fixation including the fractured vertebral is satisfactory in maintaining the deformity correction for the patients with severe osteoporotic burst fractures, the average amount of correction loss of the kyphotic was 2.0 degrees 15 months post-operation, and there were no signs of hardware pull-out, however, the reconstruction of the fractured vertebral was unacceptable, the height of the vertebral just increased from 35 and 40% to 70% in the anterior and middle portion [19]. Different strategies of hybrid stabilization about the combination of cement augmentation accompanied with posterior instrumentation for the treatment for unstable osteoporotic burst fractures have been reported, most of the studies demonstrated satisfactory clinical and radiological results, however, loss of reduction was unavoidable, the rang can be high to 4.6° to 23°, the causes of reduction appear to be the structural and mechanical deficiency of the anterior-middle columns [20, 21]. Recent study reported that the superior disc adjacent to the fractured vertebral body and the central part of the fractured vertebral body seem to be responsible for the majority of reduction loss, and this might be reduced by optimal cement technique and cement positioning between the upper and middle third of center and the anterior third of the fractured vertebral body [22].

Interestingly, height decreasing of the central part of the fractured vertebral body was limited in the pressnt study, the middle height of the fractured vertebral was maintained, it was just 2% lost at the final follow-up compared to post-operation, we consider the sequence of fracture reduction by screw-rod distraction and KP might be the contributing factor for this phenomena. Fracture reduction by balloon inflation and cement infusion was always the first stage for the past studies, while screw-rod fixation as supplementation was the second stage [13, 15, 21], however, the sequence in the present was reversed as described in the surgical technique, fracture reduction was achieved by screw-rod distraction firstly. The ability of fracture reduction is more powerful by screw-rod distraction compared to KP, as the posterior longitudinal ligament can be propped open to enhance the strength of the posterior wall by letting the retropulsed bony fragment fall in its place [23]. Meanwhile, although the balloon involved in the KP technique has a certain role in fracture reduction, scholars pointed out that KP can not achieve a satisfactory reduction effect on a compression of more than 80% [24]. Additionally, if KP is performed at the first stage, there has the following defect, fracture colud not reduced further more by screew-rod distraction if cement solidified, and the risk of screw pull out increased, while if the cement is not solidified before fracture reduction by screw-rod distraction, there would exist some space between the bone and cement, that would results in progressive vertebral collapse as there is no upholder. Although, repeats one side rod installation process looks like boring, but it’s not time consuming for an experienced surgeon.

The safety of PSFFV combined with KP is confirmed by the postoperative radiographs and there is no neurological complications, however, screw related risks and cement leakage is not devoid, and 8 patients in this series involved cement leakage, fortunately, no intracanal leakage is observed. To avoid the risk of screw malposition, the pedicle must be carefully probed in all 4 quadrants, especially for the fractured vertebral. Cement instillation should under constant fluoroscopy, which had to be stopped if the cement leaked into any extraosseous space or got close to the posterior aspect of the vertebral body.

There are some limitations concerning our study. It is a retrospective study, the number of patients is limited, the follow-up time is short, and there is a lack of control group comparing other possible surgical approaches, the stratigegy of anti-osteoporosis is not union postoperatively. Randomized controlled trials should be conducted to verify its safety and efficacy in future studies.

## Conclusion

PSFFV combined with KP is a reliable and safe procedure with satisfactory clinical and radiological results for the treatment of unstable thoracolumbar osteoporotic burst fracture.

## Data Availability

Please contact the corresponding author for data request.

## Abbreviations

PSFFV: Posterior short segment fixation including the fractured vertebra
VP: Vertebroplasty
KP: Kyphoplasty
VAS: Visual analogue scale
ODI: Oswestry disability ndex
DGOU: German Society for Orthopaedics and Trauma
OVFs: Osteoporotic vertebral fractures
